# P-1393. TikTok Fact or Fiction: Dissecting Misinformation about STIs in the Digital Landscape

**DOI:** 10.1093/ofid/ofae631.1569

**Published:** 2025-01-29

**Authors:** Marissa Nicolas, Jonathan E Pavia, Joseph Patrik Hornak

**Affiliations:** University of Texas Medical Branch John Sealy School of Medicine, Galveston, Texas; University of Texas Medical branch, Leauge City, Texas; University of Texas Medical Branch, Galveston, Texas

## Abstract

**Background:**

With over 1 billion TikTok users, the platform has seen an unprecedented influx of content. Studies have noted a significant shift in TikTok's role as a source of medical information. While some creators utilize the platform to disseminate scientifically supported content, others unintentionally or deliberately propagate misinformation. Thus, navigating the misinformation surrounding sexually transmitted infections (STIs) becomes crucial for effective infection control.

**Methods:**

Forty TikTok's were analyzed covering the CDC’s eight most common STIs: Gonorrhea, Chlamydia, Syphilis, HIV, Hepatitis B, Trichomonas, HSV, and HPV. Inclusion criteria required English-language videos with >10K likes posted after 2020. Exclusions comprised content from medical professionals, pharmaceutical companies, and non-educational videos. Two graders assessed video accuracy on a scale from 0 being completely accurate to 4 being completely inaccurate using CDC guidelines as reference. A weighted Cohen's kappa score was calculated to determine inter-rater agreement. The two grades for each video were averaged and used to calculate the results.

**Results:**

The forty videos analyzed had an average like-and-share count of 139K and 7.5K, respectively. The average TikTok score was 1.325 indicating that in general, content was not free of inaccuracies. In fact, 65% of the TikTok's analyzed contained at least one inaccuracy and about 28% were graded as mostly inaccurate (score > 2). The top three topics covered were STI transmission, symptoms, and treatment. Among these, 78% of treatment and 69% of transmission videos contained inaccurate information. Further, 44% of videos regarding STI treatment obtained a score of 4 indicating complete inaccuracy. The kappa coefficient was 0.555 indicating moderate agreement between graders.

**Conclusion:**

Today access to educational content is as easy as opening TikTok. Expanding access to STI education has potential to improve public health, but viewers must proceed with caution. Our study found that misinformation is common on TikTok, especially surrounding STI treatment and transmission. Misinformation can fuel stigmas on this sensitive topic, create barriers to those seeking proper care for STIs, and ultimately can lead to poor public health outcomes.

**Disclosures:**

**Joseph Patrik Hornak, MD**, Innoviva Specialty Therapeutics: Advisor/Consultant|Innoviva Specialty Therapeutics: Advisor/Consultant

